# What Underlies State Government Performance in Scaling Family Planning Programming? A Study of The Challenge Initiative State Partnerships in Nigeria

**DOI:** 10.9745/GHSP-D-22-00228

**Published:** 2024-05-21

**Authors:** Oluwayemisi Denike Ishola, Sarah Jane Holcombe, Andrea Ferrand, Lekan Ajijola, Nneoma Nonyelum Anieto, Victor Igharo

**Affiliations:** a The Challenge Initiative, Nigeria Hub, Johns Hopkins Center for Communication Programs, Abuja, Nigeria.; b The Challenge Initiative, Johns Hopkins Bloomberg School of Public Health, Baltimore, MD, USA.

## Abstract

**Introduction:**

Relatively few studies rigorously examine the factors associated with health systems strengthening and scaling of interventions at subnational government levels. We aim to examine how The Challenge Initiative (TCI) coaches subnational (state government) actors to scale proven family planning and adolescent and youth sexual and reproductive health approaches rapidly and sustainably through public health systems to respond to unmet need among the urban poor.

**Methods:**

This mixed-methods comparative case study draws on 32 semistructured interviews with subnational government leaders and managers, nongovernmental organization leaders, and TCI Nigeria staff, triangulated with project records and government health management information system (HMIS) data. Adapting the Consolidated Framework for Implementation Research (CFIR), we contrast experience across 2 higher-performing states and 1 lower-performing state (identified through HMIS data and selected health systems strengthening criteria from 13 states) to identify modifiable factors linked with successful adoption and implementation of interventions and note lessons for supporting scale-up.

**Results:**

Informants reported that several TCI strategies overlapping with CFIR were critical to states’ successful adoption and sustainment of interventions, most prominently external champions’ contributions and strengthened state planning and coordination, especially in higher-performing states. Government stakeholders institutionalized new interventions through their annual operational plans. Higher-performing states incorporated mutually reinforcing interventions (including service delivery, demand generation, and advocacy). Although informants generally expressed confidence that newly introduced service delivery interventions would be sustained beyond donor support, they had concerns about government financing of demand-side social and behavior change work.

**Conclusion:**

As political and managerial factors, even more than technical factors, were most linked with successful adoption and scale-up, these processes and systems should be assessed and prioritized from the start. Government leaders, TCI coaches, and other stakeholders can use these findings to shape similar initiatives to sustainably scale social service interventions.

## Abstract (precis)

This study generated insights for government partners as they expand and institutionalize interventions introduced by intermediary scaling partners such as TCI and shows the value of strengthening the factors that best support local government scaling efforts.

## Teaser

**Key Findings:**

- Strong external champions—both traditional and religious leaders but also media-savvy policy advocates—were a key component of a successful scaling experience by building public understanding and support for FP use and holding government leaders accountable for its provision.
- Skilled and empowered state program officers, with support from internal government champions at the highest political but also agency leadership levels, spearheaded the introduction and implementation of the interventions and guided the institutionalization of interventions through existing state mechanisms.

**Key Implications:**

- Earlier and more in-depth understanding of subnational health systems can enable government stakeholders and intermediary organizations to better target interventions and to sequence support to strengthen health systems and scale up interventions.
- As political and managerial (political and financial commitment; systems strength; planning and coordination) rather than just technical (knowledge about family planning interventions) capacities are most seen as linked with successful adoption and scale-up, intermediary organizations should prioritize these processes and systems from the start.

## INTRODUCTION

Globally, more women want and seek access to contraception than can obtain it, particularly in low- and middle-income countries.^1^^–^^3^ In Nigeria, low levels of modern contraceptive prevalence (mCPR) (12%) coupled with women’s high unmet need for family planning (FP) (19%)^4^^–^^6^ have prompted calls for both supply-side interventions that establish service access as well as support for strengthened social norms for FP use.^7^^,^^8^ Nigeria’s performance in meeting women’s FP and other maternal and reproductive health needs significantly lags behind other sub-Saharan African countries, a discouraging result given that it is on track to be the world’s third most populous country by 2050.^9^ Nigeria’s challenges are in meeting the needs of increasingly urban and young populations, particularly those who are poor. More than half of Nigeria’s more than 212 million residents now live in urban areas whose growth continues to outstrip that of the country as a whole (4.23% versus 2.59%), with urban populations expected to double by 2037.^10^

At the national level, Nigeria has instituted several policies and initiatives, including the 2011 Primary Health Care Under One Roof (PHCUOR) policy to strengthen health systems and programming, which includes FP. Nigeria has officially decentralized governance, including for health, down to the local government area (LGA). However, management and services at LGA levels often remain weakly implemented or aspirational, with state governments continuing to manage policy and programming.^11^^–^^13^ In Nigeria, the practical adoption and implementation of health interventions and services occur at LGA levels and are managed by the state.

However, subnational scaling processes are understudied in Nigeria^5^^,^^11^^,^^14^ as in other low- and middle-income countries.^15^ Scaling access to quality FP and adolescent and youth sexual and reproductive health (AYSRH) programming through local government health systems is an important way to address reproductive health needs, particularly of lower-income women.^16^^–^^18^ Successful scale-up of interventions by local governments can help avoid the routine pitfalls of small and intensive pilot projects that fail to serve substantial populations or to be sustained.^19^^–^^25^ Although the private sector plays a key role, governments are best positioned to provide a balanced complement of affordable FP options, particularly for low-income women.^16^^,^^26^ Strengthening government systems at the policy, managerial, and technical levels is pivotal to improving program outcomes.^27^

Successful scale-up of interventions by local governments can help avoid the routine pitfalls of small and intensive pilot projects that fail to serve substantial populations or to be sustained.

Scaling strategies have typically revealed a heavy focus on the steps and sequence of scale-up.^22^^,^^23^ Much research on the diffusion of innovations has also featured straightforward product-based innovations adopted by individuals whose use is spread through imitation.^28^ As key success factors for scaling up, Milat et al. identify several technical factors, including having a well-defined scale-up strategy, tailoring the scale-up approach to the local context, establishing monitoring and evaluation systems, conducting costing and economic modeling of intervention approaches, systematically using evidence, and having infrastructure to support implementation, as well as several managerial and political factors, including engaging a range of implementers and the target community; using participatory approaches; having strong leadership and champions, political will, and strong advocacy.^29^ More recently, the critical role of systems change to sustainably scale up complex interventions is gaining increased attention.^30^ Finally, selected voices from the health systems strengthening field have called for more attention to management capacity, particularly in light of growing investments in this area.^27^^,^^31^

We examine factors that were noted as having a facilitating, hindering, or mixed influence on subnational implementation and institutionalization of packages of high-impact FP and AYSRH interventions (HIIs), with a focus on The Challenge Initiative (TCI), a scaling platform. Starting from the premise that public health programming and results can only be strengthened and sustained if they are aligned with state governments’ objectives and commitments,^32^ this study addresses the following questions.

- What have been the key facilitating factors associated with states’ adoption, implementation, and institutionalization of FP intervention packages?
- What roles can an intermediary organization play to amplify these facilitating factors?

Scaling intermediary organizations, such as TCI, support governments to adopt and implement innovations, for example, through initial planning and fundraising, brokering among stakeholders, and providing support for change management and measurement, rather than directly implementing such interventions themselves.^33^^,^^34^

The findings may strengthen our understanding of scaling success factors and inform the broader communities of government leaders and managers, experts on scaling interventions, and technical assistance providers that seek to sustainably scale social service interventions with broad impact.

### TCI’s Demand-Driven Model and Operationalization in Nigeria

TCI’s model for scaling incorporates practices shown in the literature to be effective in adopting and expanding the implementation of interventions, as well as lessons from the demonstrated success of its precursor, the Urban Reproductive Health Initiative (URHI), implemented in India, Kenya, Nigeria, and Senegal (2010–2015).^35^^–^^37^ Since 2016 in Nigeria, TCI has worked as a scaling intermediary organization to support states to strengthen their health systems and implement HIIs in FP and AYSRH, leveraging their existing institutions and governance processes.

TCI coaches subnational actors, including state and LGA leaders, managers, and community stakeholders, to rapidly and sustainably scale HIIs for the urban poor. This demand-driven model starts with state governments who apply to partner with TCI and demonstrate both political and financial commitment to meet their own FP and AYSRH goals—subnational demonstration of effort.^38^ State governments decide which HIIs to adopt and draw on technical and managerial coaching and gap funding from TCI to plan and increase government capacity to implement, coordinate, and institutionalize these interventions. As an intermediary, TCI supports state governments to expand the implementation of interventions in new locations (horizontal scaling) and institutionalize them through government policies, budgets, and procedures (vertical scaling). After 5 years, 13 Nigerian state governments have partnered with TCI, and their health systems have served an estimated 548,609 additional FP users.

TCI’s demand-driven model starts with state governments who apply to partner with TCI and demonstrate both political and financial commitment to meet their own FP and AYSRH goals—subnational demonstration of effort.

TCI Nigeria is led by the Johns Hopkins Center for Communication Programs, in partnership with the Gates Institute for Population and Reproductive Health at the Johns Hopkins Bloomberg School of Public Health, which also supports 5 other regional hubs globally. TCI Nigeria uses multiple reinforcing strategies to support state governments to adopt and institutionalize HIIs into their public health delivery systems across the areas of service delivery, demand generation, advocacy, and monitoring and evaluation. These strategies include fostering enduring advocacy and accountability, reinforcing state leadership, management and coordination, coaching state partners, and strengthening states’ data quality and use.

## METHODS

### Study Design and Population

This is a mixed methods comparative case study of the factors associated with successful state government scale-up of high-impact FP programming. Case studies are intended to study phenomena amidst real-world conditions that are not readily separable from their contexts and to explore questions of how and why events occur,^39^ and thus help reveal complex and real-life scaling processes and factors that theories may not yet address.

This study draws on the Consolidated Framework on Implementation Research (CFIR) to examine factors seen as facilitating or hindering subnational implementation and institutionalization of packages of FP HIIs with TCI support. CFIR aggregates factors shown through research to be associated with effective adoption and sustained use of evidence-based interventions,^40^ including those from diffusion of innovations theory and systematic cross-disciplinary reviews.^19^^,^^28^ CFIR has been used widely to study adoption of innovations in high-income country health systems^41^ and increasingly in low-income country health systems.^42^^–^^45^

CFIR organizes 39 factors within 5 domains that are recognized drivers of successful adoption and scale-up of new interventions: (1) intervention characteristics, (2) outer setting (i.e., national political and social environments); (3) inner setting (i.e., the state and LGA health system adopting the interventions); (4) characteristics of individuals (adopting and implementing the innovations); and the (5) process (of adoption and scale-up). We described each CFIR construct using language and examples relevant to the Nigerian context. Under the executing factor in CFIR’s process domain, we added 5 subfactors representing TCI’s capacity-building strategies: coordination, improvement of data quality and use, demonstration strategies, integration, and institutionalization (Supplement 1 includes details on new and previous factor definitions).

The primary unit of analysis for this research is the Nigerian state government, which determines policy and programming at LGA levels. Using a 2-stage process, we selected 3 of the 13 states partnering with TCI in June 2020. First, we identified 2 pools of higher- (10) and lower- (3) performing states by ranking their growth in FP client service volume. First-stage inclusion criteria were state governments that had at least 24 full months of active partnership with TCI as of June 2020 and that were among those state governments with the highest (or lowest) growth in FP client service volume. Next, from these 2 pools, we selected 2 higher (States A and B) and 1 lower-performing (State C) states (Table 1).^46^^–^^48^ Second-stage inclusion criteria were that states had received substantive TCI coaching support and financial investment at comparable levels to other TCI partner states. The states were anonymized to allow respondents to speak candidly and not compromise their confidentiality. None of the 3 states in this study participated in the Nigerian URHI (NURHI), TCI’s predecessor. TCI coaching in State B started 6 months after coaching in the other 2 states.

**TABLE 1:** Nigerian States That Implemented TCI’s High-Impact FP and AYSRH Interventions

	**Population(Millions)**	**Total FertilityRate**	**UnmetNeed, %**	**mCPR(MarriedWomen), %**	**CurrentlyMarried WomenAged 15–19Years, %**	**BirthsAttended bySkilledProviders, %**	**FemaleLiteracyRate, %**	**Per CapitaGDP,US$ (2007)**	**LGAs, No. (%)Supportedby TCI**
State A	4.7	7.2	21	5	66	22	26	983	20 (50)
State B	3.2	4.7	20	21	12	43	53	1,587	17 (53)
State C	4.1	4.4	24	13	7	67	77	3,990	25 (52)
Nigeria	206.1	5.3	19	12	29	43	53	2,097	774

Abbreviations: AYSRH, adolescent and youth sexual and reproductive health; FP, family planning; GDP, gross domestic product; LGA, local government area; mCPR, modern contraceptive prevalence rate; TCI, The Challenge Initiative.

Source: Nigeria Demographic Health Survey, State of States factsheet, and National Bureau of Statistics.^46^^–^^48^

Higher-performing states are defined by greater growth in FP client volume using June 2020 data and increased funding commitments and expenditures for FP programs. States that recorded a decline or more limited increase in these indicators were categorized as lower-performing states.

### Data Sources and Collection

Data sources include 32 semistructured interviews with state and LGA leaders and managers in the 3 states, nongovernmental organization informants, and TCI staff, as well as TCI project records and government HMIS data.

Interview guides included open-ended questions based on CFIR domains and constructs, particularly CFIR Process domain factors that intermediary organizations, such as TCI, can more readily influence to encourage government uptake and implementation of interventions. An initial question asked informants to identify factors they saw as best explaining their state’s progress in implementing and scaling FP interventions introduced by TCI. Subsequent questions covered planning, engaging, executing, reflecting, and evaluating activities. We refined the interview guides to ensure they used language relevant to the Nigerian context and addressed topics about which interviewees would have expertise (Supplement 2 includes a sample interview guide).

We purposively selected key informants to interview based on their familiarity with state and LGA government management of work to adopt, expand, and strengthen FP interventions: 21 state and LGA managers and leaders, 3 external informants from civil society organizations familiar with FP programming, and 8 TCI staff, including 5 Abuja-based and 3 state-based coaches (Table 2). To reduce bias, the Baltimore researcher interviewed external key informants and TCI Abuja- and state-based staff and researchers interviewed state and LGA informants with whom they had no or minor previous contact. As all interviewees had postsecondary education in English, all interviews were in English. Because of the COVID-19 pandemic restrictions, all interviews were conducted and recorded virtually over Zoom or cell phones and were transcribed verbatim. Immediately after each interview, the interviewer or notetaker took notes in a standardized format to capture key interview context and content highlights.^49^^,^^50^

**TABLE 2:** Key Informants Interviewed in Three Nigerian States

** **	Government (State and LGALeaders and Managers)	External (CSO,Religious Leaders)	**TCI Abuja Staff**	**TCI State-LevelCoaches**	**Total**
State A	7	1			8
State B	7	1			8
State C	7	1			8
TCI			5	3	8
**TOTAL**	21	3	5	3	32

Abbreviations: CSO, civil society organization; LGA, local government area; TCI, The Challenge Initiative.

We also used quantitative data from routine TCI program records that included monthly state data on uptake of FP services retrieved from the national District Health Information System (DHIS2), monthly state data on implementation of HIIs (service delivery, demand generation, and advocacy), and quarterly data on health systems readiness from state performance and quality review exercises. Most indicators in Table 3 assessed progress from the start of a state’s implementation of its partnership with TCI through June 2021.

**TABLE 3.** Indicators of Health Systems Strength in Higher- and Lower-Performing States in Nigeria, Through June 2021^a^

	**CFIR Factors and Subfactors**						
	ServiceDelivery	Political andFinancialCommitment	Planning andGuidance	Coordination	ExternalChampions	**Systems Strength and Readiness**	
	**Growth inFP ClientVolume** ^b^ **, No. (%)**	**State BudgetLine Item forFP Disbursed,US$ (%)**	**HIIs Includedin State AOP** ^c^ **, No.**	**State-LedCoordinationPlatforms RegularlyOperating, No.**	**Advocacy GroupsConstituted, Registered,Operating Independentof TCI**	**FacilitiesReportingContraceptiveStock-outs, %** ^d^	**FacilitiesReportingFP/AYSRH Datain Last 12Months, %(Lowest)** ^d^
State A	+46,923 (+91)	390,385 (79)	9 (SD, DG,Advocacy,and MEL)	7 State TWGs, all includeLGA participation	ACG, IFF^e^	>10	70–90
State B	+32,683 (+15)	506,622 (126)	7 (SD, DG,Advocacy)	7 State TWGs, all include LGAparticipation; Partner CoordinationPlatform	ACG, IFF^e^	<10	>90
State C	+22,270 (+50)	187,010 (58)	4 (SD, MEL)	1 State Reproductive Health TWGincludes LGA participation; *StateTechnical Advisory Committee*^e^	ACG^e^, IFF^e^	>10	50–70

Abbreviations: ACG, advocacy core group; AOP, annual operational plan; AYSRH, adolescent and youth sexual and reproductive health; DG, demand generation; FP, family planning; IFF, interfaith forum; LGA, local government area; MEL, monitoring, evaluation, and learning; SD, service delivery; TCI, The Challenge Initiative; TWG, technical working group.

^a^Unless otherwise noted, all data are from start of states' TCI partnership implementation through June 2021.

^b^From government service statistics (health management information system).

^c^From most recent state Annual Operational Plan/budget: HIIs included are implemented across all state LGAs.

^d^Data from states’ quarterly review of performance.

^e^Not independently constituted.

### Data Management and Analysis

Transcripts were reviewed twice for completeness and clarifications were obtained from interviewers about any unclear text. All interview transcripts were imported into Dedoose 12.6 for coding and analysis. Codes were primarily created in advance based on CFIR domains and factors as adapted to the Nigeria context and TCI support strategies as explained above (Supplement 1 includes the codebook). We coded for all domains and factors, focusing particularly on process domain factors most relevant to TCI’s intermediary support to states for intervention scale-up.

For each domain of our adapted framework (Supplement 3), we present findings (illustrative quotations from interviews triangulated with related project record findings) exploring how the factors and subfactors facilitate a supportive enabling environment for intervention uptake as well as institutionalization. We finish by presenting summary results comparing states as to whether each prioritized CFIR factor was perceived as acting as a facilitator or a barrier to state government’s adoption and scale-up of interventions introduced by TCI. To reduce and organize the data to carry out cross-state analysis of patterns of barriers and facilitators, we first assigned “valence” or influence to coded excerpts. We assigned a positive (or facilitating) influence to factors if informants overwhelmingly viewed them both as aiding uptake of FP interventions in general but also as having specifically contributed to successful adoption and implementation of interventions in their state. We assigned factors either a mixed or negative influence if informants viewed them as having either an ambiguous or negative influence on the uptake and implementation of interventions in their state, respectively.

### Ethical Approval

This research was deemed exempt from full review by the Institutional Review Board at Johns Hopkins Bloomberg School of Public Health and is consistent with international standards for the ethical conduct of research. Key informant participation was voluntary, confidential, and uncompensated, and no personal information was collected except as related to the normal course of informants’ work. Key informants gave their verbal consent to participate. Guidelines from the Consolidated Criteria for Reporting Qualitative Research structured the reporting for this study.

## RESULTS

We review findings for each of the 5 adapted CFIR domains by presenting illustrative quotations and contextualizing and triangulating these interview findings with program data, including selected measures of health systems strength gathered over the course of states’ partnership with TCI. We also include how and whether informants’ perspectives align with health systems strengthening results and present results from the higher and lower-performing states (Table 3). States A and B not only had greater absolute growth in FP client volume than State C but also had greater absolute amounts and percentages of funds committed and released to finance FP programming. Further, the 2 higher-performing states incorporated more HIIs into their annual operational plans (AOPs) compared to the lower-performing state (9 and 7, respectively, versus 4 HIIs) and have more and better functioning state coordination bodies to manage FP programming that bridge from the state to the LGA operational levels (e.g., technical working groups [TWGs]). They also have more solid accountability mechanisms in place: not only do religious and traditional leaders regularly act as external champions at LGA levels but also advocacy core groups (ACGs) are now independently registered and active in health domains beyond FP. In contrast, while the ACG is active in State C, it is not registered and operates with greater support from TCI. Finally, the systems supporting FP service delivery (HMIS reporting of FP services and contraceptive commodity supply) are stronger in the 2 higher-performing states.

States A and B not only had greater absolute growth in FP client volume than State C but also had greater absolute amounts and percentages of funds committed and released to finance FP programming.

Table 4 summarizes Nigeria-specific facilitators and barriers by CFIR domain that we will discuss in more detail. Unless noted, all quotations are from state or local government staff or from state-based Nigerian external nongovernmental organization informants.

**TABLE 4.** Facilitators and Barriers for TCI Intervention Expansion and Institutionalization, by CFIR Domain^a^

**CFIR Domain**	**Facilitators**	**Barriers**
1. Intervention Characteristics	• Track record of TCI predecessor (Urban Reproductive Health Initiative) enhanced credibility and attractiveness of TCI partnership, interventions; increased state applications to partner	
2. Outer Setting	• Interventions' alignment with national policies (Nigeria's Primary Health Care Under One Roof; task sharing, task shifting, etc.) and with national and state focus on maternal mortality • Appreciation of how TCI results enable states to draw down national results-based financing (Saving One Million Lives)^b^	• Weaker tradition of political leader and civil society volunteering for community welfare (undermined by the “resource curse” of plentiful oil revenue) (Idemudia 2012^51^); local government leaders' expectation of compensation for activities (State C)^b^ • Violence and unrest preventing travel to health facilities (especially State B) • Regular shocks to health workforce: government health worker strikes, transfers, attrition (especially State B)^b^ • Health commodity, particularly contraceptive, supply chain: concern about ability to meet new demand generated^b^
3. Inner Setting	• Past donor investments in family planning and health systems strengthening: planning, human resources, state coordination platforms, HMIS (States A and B)^b^^,^^c^ • Presence of semi-operational Primary Health Care Development Agency (especially States A and B)^b^ • Increased financial commitments: creation and size of family planning and AYSRH budget allocations and disbursements (especially States A and B)^b^^,^^c^ • Use of prioritized state institutions and processes: state AOPs, TWGs^b^^,^^c^	• Persistent battles to secure release of budgeted family planning funding (especially State C)^b^ • Weak or absent state health systems and coordinating bodies, requiring establishing and/or strengthening of systems at the same time as expanding implementation of interventions (State C)^c^ • Reliance on external implementing partners to lead state Health Partners committee^c^
4. Characteristics of Individuals	• Commitment to state ownership and leadership of intervention adoption and implementation among state government staff ^b^^,^^c^	• Expectation among some State C stakeholders that TCI staff should spearhead intervention adoption and provide commodities^b^
5. Process (of Scale-Up)		
Planning and guidance	• Use of state AOPs to adopt and institutionalize interventions: higher-performing states incorporated more and a more comprehensive set of interventions, and accordingly, more are implemented in facilities at LGA levels^b^^,c^ • AOP use triggered implementation through state TWGs, heightened attention to data and outcomes, provided roadmap for advocacy for release of budgeted family planning funding^b^ • Ready availability of detailed written and coaching guidance to state staff on how to implement high-impact interventions^b^	
Spread and uptake strategies		
Government“point-people”	• Skilled and committed government staff (state program officers) designated as responsible for managing intervention implementation^b^ • Advocacy to agency heads for funding release, using data on intervention performance, in coordination with external champions^b^	
Internal champions	• Presence of internal government champions at political, agency leadership, as well as technocratic levels: aided release of budgeted funds and helped programming survive political and other transitions^b^^,c^	• Gaps in the chain of leadership commitment (agency leadership levels) impeded release of state funding for interventions (State C)^b^^,^^c^
External champions	Institutionalized presence of independent external champions (religious and traditional leaders; ACG), who: • Increased community awareness about family planning and link those interested to services^b^ • Held state governments accountable for family planning programming through advocacy for funding, formal participation in quarterly review of state programming^b^^,^^c^ • Strengthened facilities through quality improvement teams^b^	• More infrequent contributions from external champions, more rarely at LGA levels (State C)^b^
Executing		
Coordination	Step-down and implementation of interventions through existing state coordination platforms (TWGs) helped: • Institutionalize coaching on interventions^c^ • Synchronize demand generation and service delivery activities^b^ • Galvanize and better channel local participation at LGA levels (quality improvement teams) - especially in highest-performing state^b^^,^^c^ • Coordination advocacy for financial commitments^b^^,^^c^	• Weaker and more limited presence of functioning coordination platforms (TWGs) and advocacy (IFF, ACG) in the lower performing state (State C)^b^^,^^c^
Improvement of data quality and use	• Availability and use of quality data key to strengthening programming; successful advocacy with government leaders for allocation and disbursement of funding^b^^,^^c^	
Integration	• Greater popularity and perceived cost-effectiveness and sustainability of integrated interventions; data review; supportive supervision that were more frequently deployed in States A and B^c^ • Popularity of integrated approaches in framing of family planning messaging, advocacy by religious leaders^b^^,^^c^	

Abbreviations: ACG, advocacy core group; AOP, annual operating plan; AYSRH, adolescent and youth sexual and reproductive health; HMIS, health management information system; IFF, interfaith forum; LGA, local government area; TCI, The Challenge Initiative; TWG, technical working group.

^a^Table format adapted from Callaghan-Koru et al.^45^ who reported on CFIR domains and added World Health Organization/ExpandNet terminology describing scale-up through expansion or institutionalization. If no state specified, points apply to all states.

^b^Scale-up through expansion (horizontal scaling).

^c^Scale-up through institutionalization (vertical scaling).

### Domain 1: Intervention Characteristics

This domain encompasses attributes of the HIIs introduced by TCI that influenced their adoption implementation by the government and describes the core HIIs TCI introduced, including those that the 3 states now implement across all of their LGAs, not just those where there has been direct TCI coaching support (Supplement 4). States were familiar with TCI’s model and valued the positive results that they had seen from its predecessor NURHI that had been heavily publicized to Nigerian and global public health communities.^37^ TCI received double the number of state applications relative to partnership spots available, and states applying made concrete financial and human resource commitments, speaking to the attractiveness of the FP intervention package and the perceived value of working with TCI. Notably, State B’s interest in partnering with TCI increased when its application was deferred for review in the second rather than first round of TCI partner states, resulting in its commitment of increased state funding to expand FP programming.

### Domain 2: Outer Setting

Our adapted CFIR framework broadly defines the Outer Setting domain as encompassing the Nigerian national context, including government policies and programming but also the broader health and sociocultural contexts. Several national policies and programs—most notably PHCUOR but also the task-sharing and task-shifting policy and the Saving One Million Lives initiative—motivated states to partner with TCI to strengthen primary health care systems and to implement FP interventions.

Increases in use of FP services generated through adoption of interventions introduced by TCI enabled states to draw down results-based financing through schemes such as Saving One Million Lives. State B leaders were eager to move ahead in operationalizing the 2011 PHCUOR because they had not yet fully established their state Primary Health Care Development Board.

*[T]he Executive Director of the newly gazetted Primary Health Care Development Board - came to TCI to request that TCI come to State B. And immediately he committed 5 million [Naira] and put it on the table and said, “You know what, I want family planning to work.” So the fact that there was no system was the motivation for him to run after TCI because he knew that TCI was going to help him build the system that he so earnestly desired. So, that [motivation] accelerated growth and accelerated progress in State B.* —TCI headquarters, Abuja, Nigeria

States saw in TCI the chance to build health systems strength through their prioritized bodies—the State Primary Health Care Development Authority (PHCDA) and their TWGs. Relatedly, there was growing awareness and focus on how primary health care, particularly FP, could help reduce states’ high levels of maternal mortality (“childbirth spacing” is the term for FP in most states in northern Nigeria).

*State A also had this policy of …PHCUOR in operation … that also provided that opportunity to make FP as really an integral plan of primary health care. … The State has been able to see the link between the high maternal mortality we have because we do not have many women accessing childbirth spacing services.* —TCI State A Leader

Informants mentioned barriers to scale-up stemming from national-level influences, including the chronic underfinancing of public health infrastructure and shortcomings in the national contraceptive logistics management system. At the state level, violence and insecurity in State B also hampered state implementation, including facility coaching visits.

Informants mentioned barriers to scale-up stemming from national-level influences, including chronic underfunding.

In all states, informants described a historical baseline of socially conservative cultural attitudes hindering adoption and implementation of new FP interventions. State C experienced further hurdles with community leaders’ negative attitudes toward FP but also a weaker tradition of contributing to community welfare stemming from what political scientists call the “resource curse” whereby political entities endowed with monetarily valuable natural resources (oil revenue) often find their economic development stagnates because of corruption and mismanagement and discouragement of the contributions to development from political leaders or civil society.^51^

*Because down in State C, Nigeria, here, it's not as easy as in other countries where you have a lot of people, charitable organizations, individuals who really want to contribute, who want to improve their health sector. Over here, they feel that anything going into the health, any resources, is a waste.* —State C LGA Leader

### Domain 3: Inner Setting

The inner setting is defined as the state and LGA level health system, including managers, staff, and providers in the state PHCDA, the state ministry of health, and the LGAs and facilities. Informants frequently mentioned 2 factors as being influential: systems strength/readiness and political and financial commitment.

Among informants, there was consensus on the benefits of introducing and implementing new FP interventions through existing state institutions and systems. State systems included those related to planning and coordination as well as to human resources, commodity supply, and data. However, the strength and presence of states’ systems and coordinating bodies (especially the TWGs) varied at the outset of their partnerships with TCI. There was some duplication of such systems in State A, dormancy in State B, and weakness or outright absence in State C. State B and especially State A had benefited from past donor investments in FP and health systems strengthening. States A and B had (semi) operational PHCDAs, AOPs, and TWGs that made it easier to quickly adopt and implement interventions there compared to State C where they had to be created and strengthened while interventions were being introduced and implemented.

States that made greater financial commitments and disbursements were better able to implement FP interventions. As they partnered with TCI, all states made substantive political and financial and human resource commitments for implementing FP interventions. The 2 higher-performing states also both added new budget line items for adolescent and youth reproductive health. State B spent more than it had committed through its budgeted line item for FP, and State A’s release of budgeted funds is second only to State B’s. However, State C’s releases are among the lowest of all 13 states partnering with TCI and have recently tapered off further (only 16% of funds committed were disbursed in 2020–2021) (Table 3).

States that made greater financial commitments and disbursements were better able to implement FP interventions.

Although State A and State B health leaders viewed FP as an integral part of the primary health care they were seeking to strengthen and prioritized it accordingly, State C experienced resistance to focusing on FP not just among agency and department but also LGA and ward leadership. The latter had expectations that their involvement would be compensated.

*The LGA headquarters itself, they don’t place any priority in it [family planning] because they believe money does not come out of it. There are no financial benefits, so they don’t place any priority on it. They prioritize immunization services, because the government sponsors [funds] immunization a lot, so that’s where they place their priority on.* —State C LGA Leader

### Domain 4: Characteristics of Individuals

This domain captures the attitudes and beliefs of implementers (those most involved in management and implementation) toward the interventions introduced and the support offered by TCI. TCI’s intermediary role to support government scale-up of FP interventions was new for many in state and local government who were more familiar with the standard practice of many external aid organizations who themselves lead or directly implement interventions while minimally involving the government officials in planning and implementation. Government leaders and managers in higher-performing states clearly understood and embraced their role as the ones leading and implementing change and TCI’s support through management and leadership coaching, seeing this as key for interventions and programming to be sustained.

*One thing I appreciate with TCI is that they are putting you in front. It is your own, and they are showing you the way to do it so that if they are not there you can continue to do it.* —State B LGA Leader

However, some informants from the lower-performing state instead hoped TCI would serve as a source of funding for commodities and facility construction and were not fully wedded to the idea of the state taking ownership and leading interventions. Further, TCI’s role in helping the state to establish missing state mechanisms (TWGs and AOPs) also played into some state officials’ expectation that TCI would continue to lead in the work with the state.

Nonetheless, in all 3 states, state managers commented appreciatively on how TCI’s coaching approach reinforced state staff leadership—structuring roles such that state staff led, being readily accessible at all hours, and continuing mentoring and coaching despite COVID-19—enhanced the uptake of interventions, as had TCI’s transparency about funding.

In all 3 states, state managers commented appreciatively on how TCI’s coaching approach reinforced state staff leadership and enhanced uptake of interventions.

### Domain 5: Process of Scale-Up

The process domain encompasses factors related to how government staff adopt and implement new interventions. These factors are most modifiable by and relevant to TCI’s coaching of state government staff for intervention adoption and implementation. In this domain, informants associated states’ use and institutionalization of the newly introduced interventions with 2 groups of subfactors. The first set of subfactors encompassed state planning and coordination processes through which interventions are adopted and institutionalized. These processes are spearheaded by state government point people (program officers) and supported by internal (health sector) champions. Next, external champions—prominent religious and traditional leaders and advocates from outside government—were seen as central to creating a more favorable environment for FP use and programming as well as holding governments accountable for committing and disbursing funding for FP.

#### State Planning and Guidance and Coordination Mechanisms

This first group of subfactors centered on improved state planning and coordination, most notably through states’ existing AOPs and TWGs. Further, the commitment and contribution of state government point people (state program officers), backed up by internal government champions, was understood as key to effective planning and coordination in all states but particularly in the 2 higher-performing states (States A and B).

Key TCI strategies are to support states to incorporate new evidence-based FP interventions into states’ existing AOPs and to strengthen coordination mechanisms (TWGs) to implement them. Informants in all states described how AOPs structure the work of the state PHCDA, of implementing partners, and of internal and external champions. AOPs help states introduce, finance, execute, and monitor interventions at the state and LGA levels; enable state staff to manage implementing partners’ work and resources in the state; and serve as a roadmap for internal and external advocacy with policy makers for release of budgeted funding.

**Incorporating Interventions in State Plans (AOPs) to Institutionalize Them.** Across the board, informants described states as using their AOPs to adopt HIIs on FP and AYSRH and to prioritize their financing and efforts to scale up. They noted how AOPs give guidance for the interventions to be conducted at LGA levels; however, some state government AOPs included more interventions than others. As shown in Table 3, State A and State B have incorporated a more comprehensive set of HIIs (9 and 7, respectively, for service delivery, demand generation, and advocacy; and in State A for data quality improvement) into their AOPs than State C, which only incorporated 4 HIIs related to service delivery and data quality improvement. This has resulted in more of the newly introduced HIIs being fully implemented throughout all state LGAs in the higher-performing states than in the lower-performing state.

*So the Annual Operational Plan (AOP) is now state-owned and it fosters a better direction for coordination for the Primary Health Care Board. …[T]he AOP stands out because it's the gateway to ensure that the local governments (LGAs) implement high-impact interventions. It makes provisions of funding and who does what, from day 1. So it makes us very ready for what we're set to do every year.* —State B, State Program Manager

Even potentially contentious interventions, such as programming for adolescents and youth, were successfully incorporated into the AOPs of the 2 higher-performing states (with linked budget lines) and thus institutionalized. Finally, informants viewed their state AOPs as central to equipping states with the tools to hold external implementing partners accountable for their work contributing to state goals and strategies.

**AOP Incentivization of Improved Data Quality.** Informants in all 3 states described how the incorporation of interventions in state AOPs required meaningful measurement of progress. This requirement incentivized state improvement in the quality and use of HMIS data on FP services, including enforcement of partner provision of data. State program officers, through their quarterly review meetings, were the “power users” of data on intervention results. State A informants spoke far more than those from the other 2 states of their successful efforts to improve data quality and to assess achievement of goals in their AOP.

**Operationalization of AOPs Through TWGs to Roll Out Interventions (State-LGAs-Facilities).** TWGs are the key coordinating mechanisms states use to operationalize their AOPs. Informants in all states identified strengthened government coordination capacity and the TWGs, with TCI managerial coaching, as central to state adoption and implementation of new interventions at LGA levels. Intervention adoption cascades from state to LGA levels to facilities through LGA representatives in state TWGs. Informants described how states have strengthened and institutionalized coaching on intervention uptake as state TWG members coach LGA coordinators who in turn bring the new intervention to facility and ward levels through ongoing supportive supervision rather than more costly off-site trainings or headquarters-facility visits.

*We have more of that coordination between the state, LGAs, and the service delivery points, and we have now established mentoring and coaching, on-the-job training, as the need arises. …. We will continue to do (it), because we have discovered working with TCI we could use minimal resources to achieve more and it’s not all the time that we must move from the headquarters to the grassroots (LGA levels).* —State B, State Leader

Higher-performing states have a greater number of regularly functioning TWGs than the lower-performing state, including those incorporating LGA participation (Table 3). Because significant funding for the health sector typically comes from foreign sources and flows through external implementing partners, state-run TWGs actively managing these implementing partners were seen as key for full state management of implementation. However, state partner coordination meetings in State C were only held when there was direct funding from implementing partners.

### Execution

#### Strengthened or New Coordination Between Service Delivery and Demand Generation Interventions

State informants noted how the building of more formalized working ties between those working on health education (demand generation) and on service delivery at state and LGA levels resulted in increased service delivery, especially in higher-performing states. Community-based health education work was not previously explicitly linked to service delivery and did not necessarily include FP. Effective coordination of social mobilization and service delivery has drawn on improved data: use of client referral cards that reveal impacts of social mobilization activities on use of FP services, particularly in the higher-performing states. State monitoring and evaluation officers described increased use of data internally to coordinate demand generation and service delivery and to improve the service quality by targeting demand generation efforts, allocating service providers, locating and remedying stockouts, and identifying and sharing facilities’ successful strategies to improve performance.

Informants noted how the building of more formalized working ties between those working on health education (demand generation) and on service delivery at state and LGA levels resulted in increased service delivery, especially in higher-performing states.

### Spread and Uptake Strategies

#### Empowered, Committed State Government “Point People”

Informants underlined the linchpin role of designated state program officers in ensuring that state planning, and particularly coordination, activities take place effectively. In all 3 states, informants identified the state government as having formally designated state program officers within the PHCDA as responsible for managing the rollout of new FP interventions. State A informants described having particularly strong state program officers who have grown in their commitment and confidence.

*It was difficult for the state folks to actually accept that this family planning programming is their responsibility, not to look at it from the lens that this is a partner intervention. Now, over time, the state has been able to bring on board very well-versed, very well-trained hard-working program managers (officers). … So those program managers are actually cardinal to the sustainability of any program.* —State A, State Leader

Informants also pointed to these state program officers as having assumed a critical internal role in advocating for funding through data use and collaboration with external champions. State program officers in all states saw themselves as having taken on a central role in advocating internally for FP funding, as well as in launching new interventions. This role required coordinating across departments to assemble and share program data for advocacy but also collaboration with external champions (e.g., ACGs, traditional and religious leaders) to advocate to superiors for release of funding. TCI’s coaching has given them the managerial and advocacy skills to do this.

*So, when TCI came in, they showed us how it is done differently by involving key stakeholders in the community or in the state as a whole. With the help of the advocates who are advocating for childbirth spacing funding in the state, whereby we have traditional leaders, religious leaders, women leaders, youths, CSOs, journalists, who are now our voice. Because as a (state) program officer, there is a limit to what you can do even if it is what you want. … But TCI built our capacity on how to get things done, on how to get the government to commit to childbirth spacing in the state.* —State A, State Manager

#### Internal (Health Sector) Champions and Program Funding and Continuity

Informants in all states emphasized that internal champions were needed at multiple levels: state political leadership (governors and governors’ wives), agency heads, as well as at state and local government management levels. These champions were seen as facilitating intervention financing, adoption, and implementation and as helping FP programming weather transitions in state political leadership. In both States A and B, informants emphasized agency heads’ commitment, connections, and action to persuade responsive state political leaders, which they saw as key to sustainability.

Internal champions were seen as facilitating intervention financing, adoption, and implementation and as helping FP programming weather transitions in state political leadership.

*For the internal champions, we have people like the Commissioner for Health who ensures that the budget for family planning is prioritized. We have our Executive Secretary who can go to the length of going to the governor straight, so all these, to us we look at them as our champions.* —State B, State Manager

However, State C informants’ assessment of weakness among internal government champions was due to perceived gaps in the chain of government prioritization of FP programming. Technocrats alone struggled to secure funding releases without the support of agency leaders, despite backing from the state’s governor.

Further, internal (government) champions are also seen as key to providing sustained support for new interventions and programming amidst state political transitions. In State A, new incoming political leaders met a united front (agency directors and program officers) and have continued support for them and FP programming. Further, TCI’s ability to quickly identify and put in place new high-level champions (including the new governor’s wife) amidst this transition was also seen as key to sustained support.

#### External FP Champions

Across all states, informants identified external champions as having been central to improving the public environment for FP. TCI has supported the launch and work of 2 sets of external champions: (1) religious and traditional leaders working within their own institutions and with their local communities, and (2) prominent state-level advocates. Their activities include conveying the value and safety of FP to individuals and communities at the state and LGA levels (religious and traditional leaders), as well as connecting those interested to the services they want (expansion). They also hold political leaders accountable for maintaining and growing commitments to FP programming and financing and thus enhancing its sustainability (institutionalization).

Informants in all states were effusively positive about the transformative benefits of public advocacy by religious leaders (Interfaith Forum members) and traditional leaders to support FP programming. These external champions were seen as having social prestige and standing and setting up a permission structure for FP use.

*Let me say that I was working in State A here … 2007-2011. And at that time, we cannot talk about birth spacing and family planning. We cannot. But when I was talking, one of the Mallams and one of the Pastors told me that they don't care about child spacing, that it is just propaganda. Now I must inform you that there is an acceptance. The mallams and the traditional rulers are the ones engaging other people in trying to explain to the public.* —State A, State Leader

In all states, even those with greater financial and political commitments, significant energy was needed to secure release of budgeted funds for FP in state AOPs. External champions, particularly those in independent ACGs launched or strengthened by TCI, were seen as critical to maintaining government focus on FP and in spurring greater state government commitment and release of funding. ACG champions used (improved) state data to show progress, justify use of past funding, and push for release of further funds.

*Despite the fact that we have the political commitment and the demonstrated willingness to do the needful, we need a third eye … to put pressure on Government to ensure that they don't play politics with it. … But when you have an independent person also putting some external pressure on the need for some of these things to be done, it helps a lot in putting the Government or this political commitment into action.* —State B CSO Leader

**Institutionalizing External Champions’ Government Accountability Efforts**. Finally, as noted earlier, the strong advocacy structures seen as increasing the sustainability of funding for FP interventions are more institutionalized in the higher-performing states. ACGs are now officially registered in the 2 higher-performing states (Table 3), and these states’ quarterly AOP review meetings formally incorporate youth and CSO representatives, as do some TWGs.

**Sustainability Through Public Demand.** Informants in State A looked to a virtuous cycle of increasingly favorable public opinion and community demand for FP services to encourage sustained government commitment to FP programming. In State A, the popularity of the integrated 72-hour clinic makeover intervention, whereby facilities are upgraded and a private area for FP consultations added, was seen as yielding political benefits for state government leaders. Communities appreciate the tangible improvements to local facilities, and thus political leaders readily saw the value of funding further such activities to be responsive to their constituents.

*Because I will tell you the 72-hour clinic makeover (intervention) was one of the things that really gingered [interested] the state. If the governor comes and does such a program or such a project, which is done within a short period of time, the people in the community are all involved and they're happy with the setting. [So they ask] “Why won’t the government replicate such activity?”* —State A, State Manager

State A conducted 20 “72-hour clinic makeovers” with TCI support and has gone on to conduct another 6 on its own.

The Figure summarizes the perceived direction of the influence of the factors on adoption and implementation of interventions and programming. Factors that were relatively more or less discussed are also indicated. Across all states and domains, informants generally viewed factors as having facilitated uptake of interventions, and most factors were viewed as having a facilitating or mixed influence. The notable exceptions are in the lower-performing state (State C) where 2 factors were seen as being outright barriers: systems strength and grasp of and attitudes toward the TCI model. Though informants viewed State C’s systems (e.g., planning, coordination, and data) as improved, they still identified them as barriers hampering uptake of interventions. Although State C informants voiced appreciation for the TCI model and interventions, they had less uniform understanding of TCI’s emphasis on state leadership and self-reliance. In all states, the adaptive management factor (deliberate decision-making processes to improve performance that adjust in light of new information and changes in the environment), while uniformly given positive valence, was not mentioned enough to conclude that it facilitated or inhibited intervention uptake. This was also true for newly added “demonstration strategy” factor (successful demonstration of use of an HII to spur its statewide uptake and implementation).

**FIGURE.**
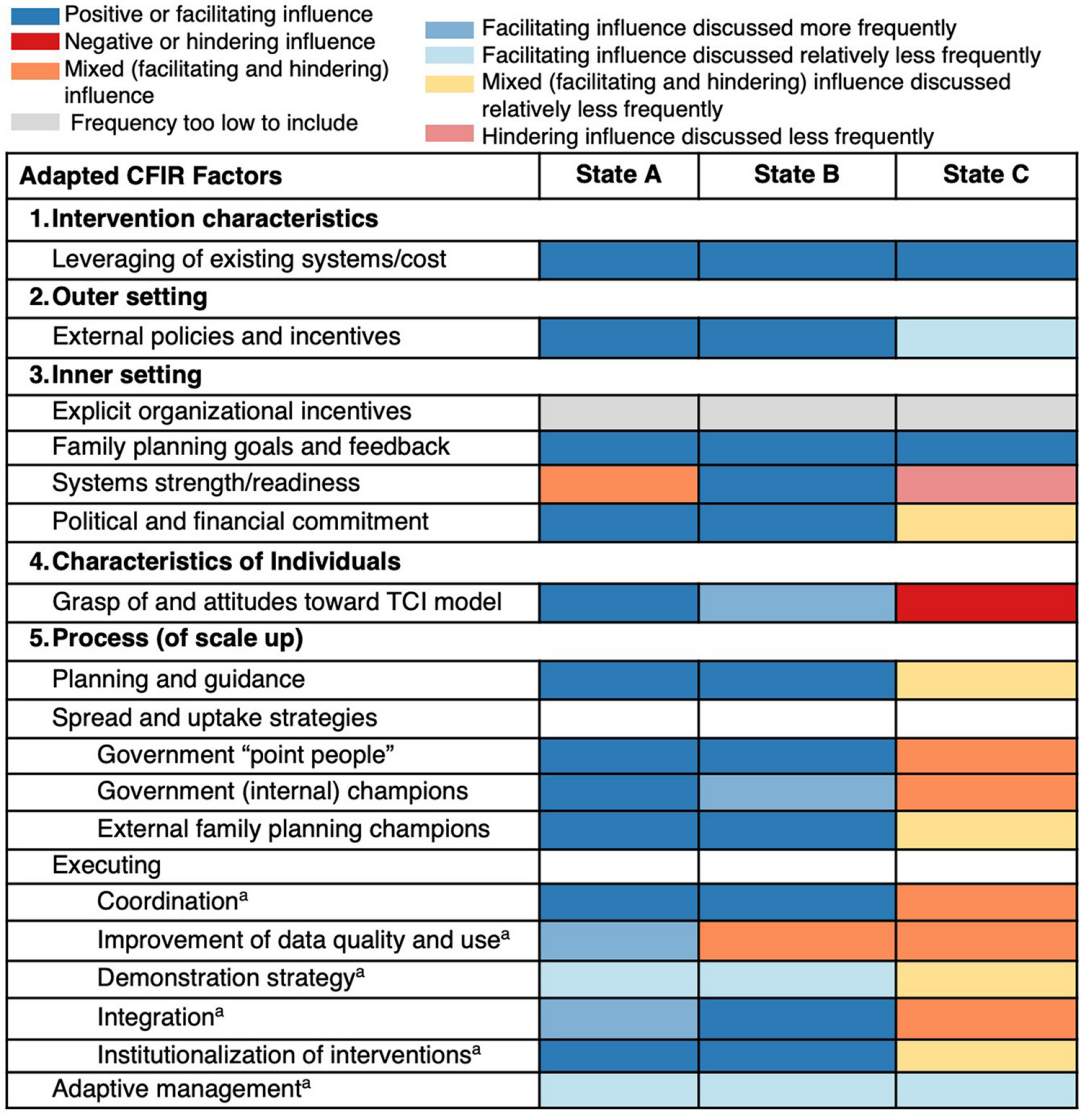
Influence of Consolidated Framework for Implementation Research Factors on the Adoption and Implementation of The Challenge Initiative’s Core High-Impact Interventions, by Nigerian State Abbreviations: CFIR, Consolidated Framework for Implementation Research; TCI, The Challenge Initiative. ^a^The Challenge Initiative scaling strategies that were added.

Unlike in the higher-performing states, informants commenting on the State C state experience more frequently described factors as having a mixed influence on uptake and implementation of interventions, and they mentioned them less frequently than did informants for other states. State C informants saw 11 of the factors examined here as having a mixed valence, while only 4 were positive and 2 were negative. By contrast, informants from the higher-performing states viewed almost all factors as facilitating adoption and scale-up.

## DISCUSSION

Deeper understanding of the drivers of success in complex programs at scale can be a daunting task. This explains why implementers often rely on a series of quantitative indicators (e.g., change in mCPR and income) to predict success. It might be expected that a state with greater financial resources and higher education levels but low mCPR and high unmet need for FP would show more progress in adopting and institutionalizing FP HIIs than states with fewer resources and weaker educational and health outcomes. However, this was not the case. We instead found that governance and leadership factors dominate explanations of states’ success, rather than the merit of the interventions, the technical capacity of the user organization, or the features of the larger national context.

We found that governance and leadership factors dominate explanations of states’ success, rather than the merit of the interventions, the technical capacity of the user organization, or the features of the larger national context.

A key component of the 2 higher-performing states’ more successful scaling experience is first that of strong external champions—both traditional and religious leaders but also media-savvy policy advocates. These champions have succeeded in both building public understanding and support for FP use and in holding government leaders accountable for its provision. They have worked through their own organizations and communities at the state and local levels because they believe in FP’s contribution to preventing maternal mortality and improving social well-being.

Next, skilled and empowered state program officers, with clear support from internal government champions at the highest political but also agency leadership levels, spearheaded the introduction and implementation of interventions. They guided the institutionalization of interventions through existing state mechanisms, namely state AOPs and TWGs. This ensured that new interventions were adopted and implemented at the LGA levels and in facilities. State program officers were also skilled in strategically allying with external champions to advocate for funding allocations and disbursements. They recognized that political and financial commitments were not fixed but rather required ongoing tending. In a fashion seldom seen in Nigeria, both higher-performing states stand out for dedicating and disbursing substantial state financing for FP programming. State B alone among TCI partner states not only made a substantial financial commitment for FP, but its expenditures exceeded its initial commitments by 126% (Table 3).

In contrast, in the lower-performing state, critical state institutions required to coordinate are weak or missing, and political and financial commitments are insufficient and unreliable. The weakness and/or absence of state planning and human resources processes, coordination mechanisms (TWGs), and data systems meant that TCI had to support the state to build or substantially strengthen these systems at the same time as the state adopted and implemented interventions. Even the support of high-level champions (the governor and his wife) was not sufficient to free up committed funds for expenditure. Internal government champions were found to be needed at multiple levels (at agency not just political and technocratic levels) to successfully access funding for programming. This state’s experience underscores the importance of "readiness" to engage with TCI and suggests that some customary measures of readiness for change and scale-up based on actual and perceived public health need (e.g., unmet need and mCPR), are not sufficiently informative. They may be less central to progress or predictive of success in scaling up.

Key findings centered on states’ readiness (systems readiness, political commitment, and actual financial disbursements) to adopt and scale interventions, with support from an intermediary partner (TCI). All states made progress in strengthening systems and expanding FP programming but from different starting points. However, through triangulation with service data, we see that states that started with more and more functional coordination mechanisms and systems (high functioning AOPs and planning processes, TWGs, etc.) were better positioned to further strengthen systems, adopt HIIs, and expand their delivery (Table 4). The state with weak or absent systems that are most critical to FP and AYSRH success had double the work: both building a minimum level of systems strength, as well as initiating and expanding intervention implementation. Notably, states’ systems readiness is less about technical ability to provide services, but rather more about state political will and governance (planning processes and coordination of implementation) and the work of external champions. However, most global conversations on development assistance and capacity-building focus on technical capacity, including technical skills related to FP skills. The global development field has long prioritized technical capacity building, often to the exclusion of managerial skills.^27^^,^^52^

States that started with more and more functional coordination mechanisms and systems were better positioned to further strengthen systems, adopt HIIs, and expand their delivery.

Finally, while the literature on scaling often makes sharp conceptual distinctions between expansion and institutionalization (horizontal and vertical scaling) of interventions, we found in practice that the 2 are necessarily intertwined at the state government level. Expanded implementation of interventions in LGA facilities necessarily flowed from their institutionalization at the state level in AOPs and through TWGs.

### Signs of Sustainability

Our findings include promising signs for sustained implementation of interventions. We see 2 notable groupings of subfactors linked with more progress in scale-up of interventions. The first set of factors relates to how evidence-based interventions and practices are introduced and institutionalized within the government. From the outset, TCI supported states to make interventions permanent by incorporating them through their existing planning and coordination processes.

A second set relates to how different types of external champions both help improve the overall environment for FP but also hold governments accountable for maintaining and growing FP interventions and programming. The institutionalization of these external accountability mechanisms, such as through registration of independent organizations (e.g., ACGs), incorporation of external champions in regular state review practices, including regular participation in functioning facility quality improvement teams in both higher-performing states, shows promise for a sustained positive feedback cycle. For example, in State A, priority for FP has continued despite a recent political transition in the state. Finally, the progress in establishing mature and collaborative “insider” advocacy processes and independent accountability structures in the higher-performing states is encouraging, although the relationships are young.

### Limitations

This study draws on a validated set of factors associated with successful widespread adoption of interventions (CFIR) and triangulates interview findings with project records and HMIS data in each of the 3 states. Informants were candid in identifying and assessing threats to sustainability of the new interventions and programming. Despite these strengths, our findings cannot demonstrate causality but only suggest that patterns seen may be transferable to other similar contexts. Further, as with any study using interviews, there is a possibility of flawed memories and statements that reflect what interviewees (government officials partnering with TCI) perceive interviewers want to hear. In addition, despite the lower-performing state’s substantially stronger overall socioeconomic status, its lower levels of investment in state infrastructure likely hindered states’ ability to scale up FP interventions and thus suggest it would be valuable to explore whether these findings align with those in other lower-performing states. Finally, while half of LGAs in each state received TCI coaching, the same numbers of TCI staff had to coach in more LGAs in the lowest-performing state (13 versus 9 or 10 LGAs), potentially “diluting” the dosage effect of TCI in State C.

## LESSONS FOR SCALING INTERMEDIARIES, GOVERNMENTS, AND FUNDERS

### More Comprehensive and Earlier Understanding and Measurement of State Systems Strength

The experience in the lower-performing state suggests that intermediaries should prioritize partnering with states with adequate systems in place and to do so, they should at the outset assess the presence and strength of state governance and data systems key for scaling up FP HIIs. This requires measurement of systems strength as well as of service outcomes to gain a fuller picture of states’ starting points and progress. Such assessment would provide a better understanding of which interventions states are equipped to adopt and when; which coordination bodies are the most critical to support to ensure intervention uptake; and how to improve sequencing, structuring, and institutionalization of intermediary coaching support. When intermediaries partner with states that are less ready, they should set expectations about the greater time and resources required to achieve results similar to those of states with greater initial systems strength.

### Need for Ongoing Cultivation of Political and Financial Commitment

The lesson from all states is that political commitment is not fixed but rather needs to be created and maintained through ongoing advocacy at multiple levels (political and managerial). Accordingly, from the outset, intermediary organizations should foster more sustainable advocacy mechanisms to keep FP interventions and programming prioritized and to ensure committed funding is disbursed.

The lesson from all states is that political commitment is not fixed but rather needs to be created and maintained through ongoing advocacy at multiple levels (political and managerial).

### Support for Champions in Moments of Crisis

Scaling intermediaries have a role to play in supporting (new) champions in times of political transition or crisis. While champions ideally need to be in place at the outset and continuously cultivated, in a crisis/transition moment such as election-related staffing transitions, the scaling intermediary group should be ready to quickly foster support among (additional) government leaders responsible for authorizing the release of budgeted funding so that there is funding continuity and expanded provision of new interventions.

### Institutionalization of Intermediary Coaching

Intermediaries can strengthen state capacity in a more sustained manner by institutionalizing coaching within existing government bodies. An example is TCI’s coaching of the state’s SBCC committees that include the state health educator and representatives from each LGA (i.e., health education officers).^53^ This strategy of providing systems-level coaching enables capacity retention even with staffing transitions and ensures a cascade of capacity building and diffusion of HIIs across the state, not just in TCI-supported LGAs.

## CONCLUSION

Subnational governments, particularly in low- and middle-income countries, have many competing priorities and limited human and financial resources. Several areas of support, most notably related to external champions, state planning, and state coordination are required to successfully introduce and maintain FP and AYSRH HIIs at the top of state government agendas. The findings here show the value of strengthening the more modifiable factors that best support local government scaling efforts—state governance structures and managerial skills—and thereby leveraging scarce resources for impact. Through use of a validated conceptual framework (CFIR) and examination of the experiences of higher-performing states and contrasting it with those of a lower-performing state, this study generated insights useful to government partners as they expand and institutionalize the use of approaches and interventions introduced by intermediary scaling partners such as TCI. While it is not possible to generalize directly from the findings here, we believe that the patterns seen here merit consideration by others trying to scale up interventions at subnational levels.

## Supplementary Material

GHSP-D-22-00228-Supplements.pdf

## Notes

**Peer Reviewed**

**First published online:** December 9, 2022.
