# Supplementary material for: What Underlies State Government Performance in Scaling Family Planning Programming? A Study of The Challenge Initiative State Partnerships in Nigeria
Source: Glob Health Sci Pract. 2024 May 21;12(Suppl 2):e2200228. doi: 10.9745/GHSP-D-22-00228 (PMC11111101; doi:10.9745/GHSP-D-22-00228)
Supplement: GHSP-D-22-00228-Supplements.pdf [file GHSP-D-22-00228-Supplements.pdf]

Supplement to: Ishola OD, Holcombe SJ, Ferrand A, Ajijola L, Anieto NN, Igharo V. What underlies state government performance in scaling family planning programming? A study of The Challenge Initiative state partnerships in Nigeria. *Glob Health Sci Pract*. 2023;11(Suppl 1):e2200228. https://doi.org/10.9745/GHSP-D-22-00228

Supplement 1: TCI CFIR codebook

(with codes described in terms of TCI interventions and State contexts)

Title	Description
**I. INTERVENTION CHARACTERISTICS**	
**Adaptability of High Impact Interventions**	Describes the extent and type of modifications/changes in the high impact interventions (HIIs Nigeria) / High Impact Approaches (HIAs India) introduced by TCI to the City/State in order to better fit and operate in the local context. For example, taking into account local administrative structures or availability of human resources. Adaptations may take place at the Hub HQ level prior to launch, at State/City levels during introduction; at facility levels). This can also apply to the TCI model for interacting with the State/city.
**Alignment with National/State Policies**	The degree to which informants perceive that The Challenge Initiative (TCI)'s interventions and programming are aligned with national and state policies and programming. In Nigeria, examples include Primary Health Care Under One Roof and the Task Sharing Task Shifting policies: Saving One Million Lives (SOML).
**Appeal of the TCI Model (HIIs/HIAs)**	Extent to which government and other respondents indicate that the TCI's model represents a desirable and impactful set of approaches. This could include enthusiasm for Business Unusual strategy (mindset shift), or for individual HIIs such as 72-hour makeover intervention, TCI-U, etc., or for other practices introduced by TCI. etc. (Perceived as helpful to existing mission of government.)
**Availability & Value of Alternatives**	Perceived availability and strength of any alternative strategies for expanding strength and reach of government family planning programming. If offered, any assessment of the relative value of TCI's programming versus that of other outside agencies. This can also include comparison of TCI's approaches with existing practices. Sibling code: Appeal of TCI Model High Impact Interventions (HIIs)
**Complexity**	The extent to which TCI's model and/or HIIs are perceived as complicated, burdensome, difficult to carry out, including whether the implementation of the individual HIIs (the stages/steps, the knowledge and human, financial and logistics resources required is seen as complex. In Nigeria, an example is how TCI works with government to simplify/streamline government data quality improvement processes (often cross-coded with integration). EXCLUSION: discussion of requirement for financial resources should NOT qualify TCI interventions to be coded with "Complexity".
**Evidence Strength & Quality**	Government stakeholder perception that TCI program offers high quality, evidence-based strategies.
**Latitude for testing**	Opportunity to test interventions on a smaller scale prior to fully rolling out across all geographies. Ex.: Scope for testing in ability to rollout in each city/LGA and then to bring learning to the next geography (city, LGA). [TCI HIIs already tested during NURHI] [Caution: is to be distinguished from, but can be co-coded with, 'Adaptability'.]
**Leveraging of existing systems (Cost)**	The extent to which implementation of the TCI model is able to capitalize on existing State and City systems, practices and programming (rather than creating new ones), thus reducing costs and enhancing the likelihood of long-term sustainability. This includes working through existing government committees and mechanisms, drawing on existing data and data systems - explicitly NOT creating new free-standing systems that cannot be maintained by government in the longer term.

Supplement to: Ishola OD, Holcombe SJ, Ferrand A, Ajijola L, Anieto NN, Igharo V. What underlies state government performance in scaling family planning programming? A study of The Challenge Initiative state partnerships in Nigeria. *Glob Health Sci Pract*. 2023;11(Suppl 1):e2200228. https://doi.org/10.9745/GHSP-D-22-00228

**II. OUTER SETTING**	
**Client's (women's) Needs**	Client (women's) needs: Awareness of and extent that individual women's (and adolescents') particular needs for family planning are recognized, understood, and prioritized by government and key supportive stakeholders (champions). In India, this can include understanding of the existing patterns of contraceptive method use, and of the needs of minority populations (in light of how TCIHC & city interventions may align or disrupt).
**Competitive Pressure**	See "Institutional rewards"
**External Policy Incentives**	Extent to which there are national/state policies and regulations that encourage the adoption or (more vigorous) implementation of TCI's FP programming (In Nigeria, examples include Primary Health Care Under One Roof; Task Sharing Task Shifting policies; SOML; India: National Urban Health Mission; RKSK, ESB). [Often co-coded with "Alignment with National/State Policies".]
**III. INNER SETTING**	
**Access to Knowledge & Information**	Availability of information on how to carry out the HIIs and the HIAs, as well as how to institutionalize them - as well as awareness of this on the part of the key government staff. This can include discussion of the utility of TCI-U and availability of TCI staff to provide technical coaching to government staff and workers. [This is linked to, but not the same as the code for "Spread/Uptake Strategies", which describes more active strategies.]
**Climate for Organizational Learning**	How welcoming the government climate is to efforts to learn, test and adopt new approaches (Previously for TCI " Whether and How TCI has been able to learn and shift gears when it encounters challenges in operationalizing its model. this is now in PROCESS/Adaptive Management code.)
**Compatibility with existing systems**	The extent to which the TCI family planning HIIs/HIAs and programming fit with (do not disrupt or replace) existing government systems and workflows (HR, service delivery, demand generation, information). Sibling to "Leveraging of Existing Systems".
**Culture**	The values and orientation of government institutions, public sector health institutions (ex. PHCDA in Nigeria; NUHM or city health programming in India) regarding adoption of change, and to strengthening family planning programming. This can also include government staff perspectives on the broader outside culture regarding family planning.
**Explicit organizational incentives**	The extent to which the government institution has a structured incentive system for individual staff to reward achievement of goals regarding strengthened family planning programming. In Nigeria, this could involve TWG monthly follow-up on service statistics; QIT meetings. This can also include reference to TCI efforts to better use existing systems to reward FP programming achievement. ["Institutional rewards for Success" will be folded into this code.]
**FP Goals & Feedback**	The extent to which family planning program goals are set, shared and known, throughout the levels of the health care system. For example, evidence in Nigeria could include discussion of Annual Operational Plans, Technical Working Groups, facility 'run charts'[See also the codes for 'Explicit Organizational Incentives' and 'Institutional Rewards for success'.]
**Institutional rewards for success**	Extent to which government leaders and personnel are in a system where they are under pressure to perform. Also, extent to which the system will reward government staff (particularly leaders) for performing well with respect to family planning programming. This can also include reference to TCI efforts to better use existing systems to reward FP programming achievement.

Supplement to: Ishola OD, Holcombe SJ, Ferrand A, Ajijola L, Anieto NN, Igharo V. What underlies state government performance in scaling family planning programming? A study of The Challenge Initiative state partnerships in Nigeria. *Glob Health Sci Pract*. 2023;11(Suppl 1):e2200228. https://doi.org/10.9745/GHSP-D-22-00228

**Organizational structure**	Typically measured quantitatively, however, presence of large institutions with formalized hierarchies. In Nigeria, this can include particular discussion of the strength of institutions and implementation at the LGA and facility levels and the status of FP programming work there. This can also involve discussion of government human resource constraints as well as discussion of government accountability (creating feedback from LGA, community levels). In Nigeria, this includes discussion of the strengthening of LGA level capacity.
**Community Engagement & Accountability**	The degree to which:<br>a) local level community members have a structured visible way to participate in local health governance structures or to have their voices heard by political and health systems or members of local level political structures are<br>b) local level officials (Nigeria: LGA and village World Development Committee);<br>c) State level political leaders perceive themselves as responding to public demand for FP availability (funding, facilities, education, services)
**Perceived Gravity of Underlying Problems**	How respondents describe the degree of seriousness of the problems that strengthened family planning programming aims to address. Discussion of the seriousness of the problems of maternal mortality, unsafe abortion, unplanned and early pregnancy, population growth, are examples of how informants may indicate their perceptions of the scope of the problem. Further, this may include discussion of how weakness of FP programming can jeopardize well-being of women and the broader population, requiring change.
**Political & Financial Commitment**	The degree to which political leaders give political and financial priority to family planning, through public statements, resource allocations, or other support, as well as see the TCI partnership as key to furthering family planning programming. This code applies to government leadership actively working to strengthen family planning programming. Examples include incorporation of budget line items for family planning as well as actual release/disbursements of funding for activities; discussion of government spending on HIIs (requires Executive and/or Legislative branch approval) implying commitment of funds and establishment of enabling policies; government leaders' public statements in support of FP, benefits of FP policies and programming, need for FP funding. [See also "Priority for FP".]
**Priority for Family Planning**	The extent to which the government assigns a high priority to family planning, in its own right (now vs. in the past), as well as relative to other health areas. [Often also coded with 'Political and Financial Commitment']
**Systems strength/ readiness**	At the start of the partnership with TCI, describing the relative (strong or weak) strength of the government systems that are necessary to manage and implement family planning and other health programs. These systems could include planning, data collection and use, partner coordination, commodity logistics, etc. For example, a health system in Nigeria has a lower level of readiness if it lacks regular workplanning processes.
**IV. CHARACTERISTICS OF INDIVIDUALS**	
**Grasp of & Attitudes toward TCI model**	Extent to which an individual respondent understands how the TCI partnership works and its goals, as well as her/his degree of support for it. If a person appears highly supportive of the model, but fails to understand a key component of it (for example, that TCI is not the implementer but rather is a coach, and that the government should be leading the implementation), then they should not be defined as supportive. [Linked codes: "Self-efficacy"; State of Implementer Commitment"]
**Self-efficacy**	An individual's belief in their own capabilities to execute courses of action to achieve implementation goals. [Linked codes: "Stage of Implementer Commitment"' "Grasp of & Attitudes toward TCI Model"]

Supplement to: Ishola OD, Holcombe SJ, Ferrand A, Ajijola L, Anieto NN, Igharo V. What underlies state government performance in scaling family planning programming? A study of The Challenge Initiative state partnerships in Nigeria. *Glob Health Sci Pract*. 2023;11(Suppl 1):e2200228. https://doi.org/10.9745/GHSP-D-22-00228

**Stage of Implementer commitment**	Degree to which government personnel and other involved individuals are aware of the HIIs and other practices introduced by TCI, committed to implementing them, and confident and capable in actually carrying out and leading the related work. This can range from government implementers who are unaware to those who are informed and deeply involved in and committed to implementation - and OWN/LEAD implementation. This code applies to instances where informants show what TCI refers to as 'mindset shift' as well as ownership of and buy-in to the TCI model and approaches. [Linked codes: "Grasp of & Attitudes toward TCI model"]
**TCI characteristics**	Description of key features of TCI staff, the ways they work to support government, and the extent to which these are seen as related to the successful strengthening of FP programming and as differentiating TCI from other agencies/implementing partners. This could include any description of TCI's coaching strategy. [Linked with PROCESS domain, including "Spread/Uptake Strategies" as well as what would have been CFIR "opinion leaders" construct].
**V. PROCESS**	
**Planning and Guidance**	Refers to initial up-front codified plans for state-TCI partnership and for implementation of high impact interventions (HIIs) introduced by TCI (through the state Expression of Interest (EOI), Program Design (PD) document, and codified interventions), and the extent to which there are explicit government annual operational plans (AOPs) incorporating interventions, as well as of detailed descriptions of how to implement them.
**Spread/Uptake Strategies**	Use of strategies for supporting government staff in the uptake of TCI-introduced practices & evidence-based interventions:
**Government Partner 'Point People'**	Whether there are people within government who have been identified to assume leadership of the partnership with TCI and to take responsibility for guidance of implementation of the new work to strengthen and expand the government's family planning programming. AND, who actually takes on leadership of implementation. In Nigeria, this could include both the appointment of a particular staff member within the Primary Health Care Development Agency (PHCDA) to serve as a TCI counterpart, or could involve strong, ongoing and regular contact and connection between senior PHCDA leaders and TCI.
**Internal (Government) Champions**	Describing the leaders within the government health system who actively work in support of strengthened FP programming. This includes Executive Secretaries of the Primary Health Care Development Agency (PHCDA), Ministry of Health, Governors, as well as other key state leaders such as Technical Working Groups (TWG) and LGA-level leaders and managers.
**External Champions**	Describing actors external to government health institutions who, either in their professional or personal roles, serve as advocates and supporters of family planning services, programs and policies. These actors can be religious leaders (Interfaith Forum members), operating at either the state or LGA levels), advocates or advocacy groups (such as Advocacy Core Groups), political leaders (such as members of state legislative bodies in Nigeria). The influence can take at least two forms: pressure directly on government to support provision of FP (budget allocations, services) OR public support offered more at community levels in support of individual use of FP services. This can also include incorporation of youth voices for accountability.
**Executing**	Carrying out or accomplishing the implementation according to plan:
**Coordination**	Strategy to mobilize and organize human and other resources vertically and horizontally across government and beyond to achieve expanded family planning programming. including through state-level planning and coordination processes in policy, service delivery, demand generation; Advocacy Core Groups; Interfaith Forums; government Technical Working Group members; coordination of outside implementing groups ('partner coordination' of international NGOs); cross-departmental groups; LGA-level facility Quality Improvement Teams (QITs) engaging the

	community and local leaders. TCI coaching is central to supporting government staff to coordinate better.
**Improvement of data quality and use**	Description of the use of data to assess whether interventions are working and to improve and/or expand management of these interventions and programs. This can include targeted sharing of results to inform decision-makers and implementers (creating a virtuous feedback cycle of data use). This can take place through mechanisms/processes such as: Nigeria: periodic TWG and QIT meetings.
**Demonstration strategy**	Description of a strategy to strengthen government ownership of and momentum of family planning programming through demonstration of results. When positive results are demonstrated, this furthers a 'virtuous cycle' of increased government support for family planning programming, and a willingness to invest more energy and resources to expand programming (e.g., use of 72-hour clinic makeover or In-reach interventions).
**Integration/ Integrated services**	Refers to joint conduct of family planning and other activities (health care, data collection and review, etc.) This represents the approach of the Nigeria Federal government (Primary Health Care Under One Roof) that is being transmitted to State governments and implemented through State Primary Health Care Development Agencies/Boards. The aim is the integration of all PHC services delivered under one authority; it implies providing two or more primary health care services on the same platform. Examples: health professionals providing ANC can also counsel on contraception; state monitoring and evaluation workers can review quality of immunization data but also family planning data in the same meetings.
**Institutionalization**	Discussion of how and the extent to which proven interventions and new government practices that have been introduced by TCI have been integrated into the regular course of how the government institutions do their work (policies, budgets, guidelines, etc.). The code also covers the extent to which family planning funding commitments and releases have been regularized. In Nigeria: discussion of AOPs, state budget line items for FP, AY, etc..
**Adaptive Management**	Where and whether TCI and/or government staff gather information to examine how well government staff (either at the facility, LGA, or State levels) are implementing or how well TCI is supporting government staff to implement, and then reflect on what steps to take to strengthen how government staff members (TCI) go about their work. Refers to whether and how TCI and government officials have been able to learn from evidence and experience and shift gears when encountering challenges or setbacks in adopting and sustaining interventions.

Supplement to: Ishola OD, Holcombe SJ, Ferrand A, Ajijola L, Anieto NN, Igharo V. What underlies state government performance in scaling family planning programming? A study of The Challenge Initiative state partnerships in Nigeria. *Glob Health Sci Pract*. 2023;11(Suppl 1):e2200228. https://doi.org/10.9745/GHSP-D-22-00228

Supplement 2 - Sample Interview guide

**KEY INFORMANT INTERVIEW GUIDE, STATE GOVERNMENT IMPLEMENTERS (Plateau & Bauchi)**	
**Interviewee** name, job title, department:	
State (LGA):	
Interviewer (& Notetaker) name(s):	
Interview date and time:	
Interview modality:	phone/internet call __ /other _________ (*circle and/or fill in one*)
Links to transcribed file & recording:	

I. ***Introduction and study purpose***

Thank you for agreeing to participate in this study. We want to understand how states in Nigeria are making childbirth spacing/family planning (and life planning for adolescents and youth) more available and accessible. We are interviewing people from a variety of stakeholder groups, particularly leaders and managers working at the State government or LGA levels or in health facilities such as yourself. We are interested in discussing how State governments work on health and childbirth spacing and your experience with TCI. Our aim is to find out how TCI can improve how it work with States to support childbirth spacing programs and to share these findings more widely.

II. ***Interview logistics***

We would like to start by introducing ourselves and providing some information about the interview.

a) Introduction to the interview team

- The Challenge Initiative (TCI) is a program funded by the Bill & Melinda Gates Foundation to support local governments to strengthen and expand family planning and adolescent reproductive health programming.
- The interview team consists of members in the U.S. with The Challenge Initiative at Johns Hopkins University and in each of TCI's four global regions, including here in Nigeria.

b) Information about the interview and request for your consent to participate

- *Length:* The interview is expected to last approximately 1 to 1.5 hours.
- *Anonymity:* We will not be linking any of the information that you share to your name without your permission. *Clarity and Consent:* Please feel free to ask any questions if you do not understand. You can end the interview at any time.
- *Notes and Consent to Record:* We will be taking notes during the interview, and I'd like to record our conversation. This will help me go back and fill in my notes more accurately. May I record our conversation? *[Wait for verbal agreement.]*

III. Interview Guide Questions

Thank you for taking time to talk with me today. Please note that there are no right or wrong answers – you are the expert. So, feel free to be as direct as possible. Before we begin, do you have any questions? [*Question and answer, as needed*]

Supplement to: Ishola OD, Holcombe SJ, Ferrand A, Ajijola L, Anieto NN, Igharo V. What underlies state government performance in scaling family planning programming? A study of The Challenge Initiative state partnerships in Nigeria. *Glob Health Sci Pract*. 2023;11(Suppl 1):e2200228. https://doi.org/10.9745/GHSP-D-22-00228

General *(Always ask this question first. This is a rapport-building question)*
I would like to begin by asking you a few background questions.
1. Can you tell me more about yourself? In particular, how long have you worked in health and on childbirth spacing, and how long have you been in your current role?

Overview Questions
2. Thinking about the past two years, what do you think are the key reasons why your State government has been able to increase availability of and access to childbirth spacing (and life planning for adolescents and youth) programming for those who want it?
Probes:
- Do you see any other reasons?
- In your opinion, what is the most important way that TCI has helped the State to strengthen childbirth spacing (life planning for adolescents and youth) programming?

3. Thinking about the past two years, has your state government made any specific efforts to strengthen programming on life planning for adolescents and youth? If yes, what are they? (If yes, probe for TCI involvement.)

INTERVENTIONS AND INTERVENORS (TCI)
State government adoption of high impact childbirth spacing (& LPAY) interventions
4. Over the past two years, what specific TCI high impact interventions for childbirth spacing (or life planning for adolescents and youth) has TCI introduced to the State? Which ones has your State adopted into its State workplan and institutionalized within its childbirth spacing program? How did this happen? (Probe only if necessary: any interventions related to service delivery? To demand generation? To advocacy? To RME?) What do you see as the results of the State's adoption of these high impact interventions?

5. Once your state government has decided to adopt a certain high impact intervention or practice (such as in-reaches, Whole Site Orientations, 72-hour Makeovers), how does it coordinate with the LGAs to implement it and incorporate it into their regular ongoing work? Please use an example of an intervention that your State has adopted. *(In other words, what are the key steps that the State should take to maximize successful LGA adoption and implementation on an ongoing basis?)*
Probe only if necessary:
- What are common challenges when State government works with LGAs to implement a new high impact intervention?
- How do State government staff try to overcome these challenges?

6. Has the State changed any high impact intervention introduced by TCI in order to have them better fit into the local context of the State and LGA? (For example, as the State designs the AOP?) If yes, please share an example.

Supplement to: Ishola OD, Holcombe SJ, Ferrand A, Ajijola L, Anieto NN, Igharo V. What underlies state government performance in scaling family planning programming? A study of The Challenge Initiative state partnerships in Nigeria. *Glob Health Sci Pract*. 2023;11(Suppl 1):e2200228. https://doi.org/10.9745/GHSP-D-22-00228

State government adoption of improved practices

7. In the past two years, has TCI helped you make any changes in your processes or systems to strengthen your childbirth spacing program? Probes if needed: For example, have you seen changes in State practices or processes such as state workplan/budgeting, financing for childbirth spacing (budget lines), partner coordination and management, stepping down of national guidelines, use of data for program management, data quality review, etc.?

Probe: If yes, can you describe how the changes took place using one of the examples just mentioned?

8. Has working with TCI changed the way you do your job? If yes, how?

Institutionalization of interventions (and practices)

These next questions are aimed at understanding how permanent the high impact interventions and processes that the State has adopted are likely to be.

9. We all know that partner programs do not last forever. If TCI ends tomorrow, which of the high impact childbirth spacing interventions that have been introduced by TCI do you think would be most likely to continue in the State over the longer term? Which ones are most likely to disappear? Why? *(Only use if needed: In other words, from your perspective, how well are the high impact childbirth spacing interventions and life planning for adolescents and youth interventions introduced by TCI integrated into the everyday operations of the State and likely to last for the longer term?)*

Intervenor (TCI) characteristics

Now I have questions about any features of TCI's way of operating/operations that may have helped in the introduction and adoption of new practices.

10. How is TCI's approach to work with the State government the same as that of other counterparts? How is it different from that of other counterparts?

11. Have your TCI counterparts (TSL) been helpful in supporting the State to expand childbirth spacing programming? If yes, how? (What are the key ways?) What could be strengthened? *(Prompt if needed:* This could be either the way they work with State colleagues or TCI counterpart characteristics.*)*

PROCESSES: Government systems and coordination processes

Now I'm going to ask you questions about specific aspects of how government works (processes, coordination mechanisms) that may relate to how States can successfully expand access and availability of childbirth spacing programming and of life planning for adolescents and youth for women and youth who want it.

Use of data for program management

12. Can you describe generally how your State uses data to manage and improve its childbirth spacing programming? What if any changes have you seen in how the State uses data over the past two years? (Probe, if needed: In other words, please describe how your State

monitors and assesses the quality and effectiveness of its childbirth spacing program, uses data to identify and address problems and select productive strategies, etc.).
Probes:

- How does the State track progress serving youth and adolescent populations? How, if at all, has age-disaggregated data been used to improve programming?
- In your opinion, how, if at all, has TCI contributed to these changes?

Coordination
13. What state planning and coordination processes are most key to managing your State's childbirth spacing progamming, including all the partners? (Such as state planning and coordination processes in policy, service delivery, demand generation; use of data for management, committees, cross-departmental groups, meetings, QITs)? Why?
Probes:

How have these processes changed over the past two years, if at all? (If not mentioned: In what ways, if any, has TCI contributed to these coordination processes?)

INTERNAL CONTEXTUAL FACTORS (State & LGA Government - priority for childbirth spacing, alignment of the innovation with the context))
Now I am going to ask you a few questions about your State government's priority for childbirth spacing.
14. How much of a priority does your State place on childbirth spacing compared to other health areas? How has this changed over the past two years? What has contributed to this change?
Probe if not mentioned: To what extent, if any, has TCI played a role in this change?

Leadership/support (see also government priority)
10. In your State, over the past two years, how important a role have FP champions or supporters played in expanding availability of and access to childbirth spacing and adolescent and youth programming? What has this role been? At what points is the support of champions most helpful?
Probes if needed:

- What about internal champions within State government? (such as support from both political leaders and technocrats: Governors, Executive Secretaries, State Coordinators, etc.)
- What about external champions? (champions from outside government such as from Interfaith coalitions; ACG members, TWG members, etc.)

'Spillover' or unexpected effects of State's partnership with TCI
These questions ask about whether you have seen any 'spillover' or unexpected effects of your State's partnership with TCI.
15. What additional or unexpected effects have you seen resulting from your State's partnership with TCI – beyond expanding access to childbirth spacing programming and to life planning for interested women and youth?

Probe: Such as on State health systems, on the State's management of other health programs, or on achieving the State's broader primary health care agenda?
(*Probes if informant does not respond*: For example, unexpected effects could include policy/guidelines changes; improved use of data for decision-making; improved service quality or facility readiness; diffusion of interventions introduced by TCI to other (non-TCI) sites; better coordination among government stakeholders and implementers; fewer stockouts; integration of services; changes in social norms/ community acceptance of FP; etc.)

CONCLUDING QUESTIONS:

16. In future, what do the LGA and the State most need to do to strengthen and grow family planning programming in the State to make it last? (If needed: what do you see as the one or two chief barriers to your LGA continuing to expand access to family planning programming on a lasting basis?)

17. Is there anything else you would like to tell us? Are we missing any points that explain how States can expand access to childbirth spacing programming on a sustainable and long-term basis?

Thank you very much again for participating in this interview. We appreciate that you have shared your valuable expertise, perspectives and time with us. We hope that we can contact you again if we need to clarify any questions.

Supplement to: Ishola OD, Holcombe SJ, Ferrand A, Ajijola L, Anieto NN, Igharo V. What underlies state government performance in scaling family planning programming? A study of The Challenge Initiative state partnerships in Nigeria. *Glob Health Sci Pract*. 2023;11(Suppl 1):e2200228. https://doi.org/10.9745/GHSP-D-22-00228

Supplement 3- Adapted Conceptual Framework for Nigeria study (adapted from Damschroder et al. 2009.

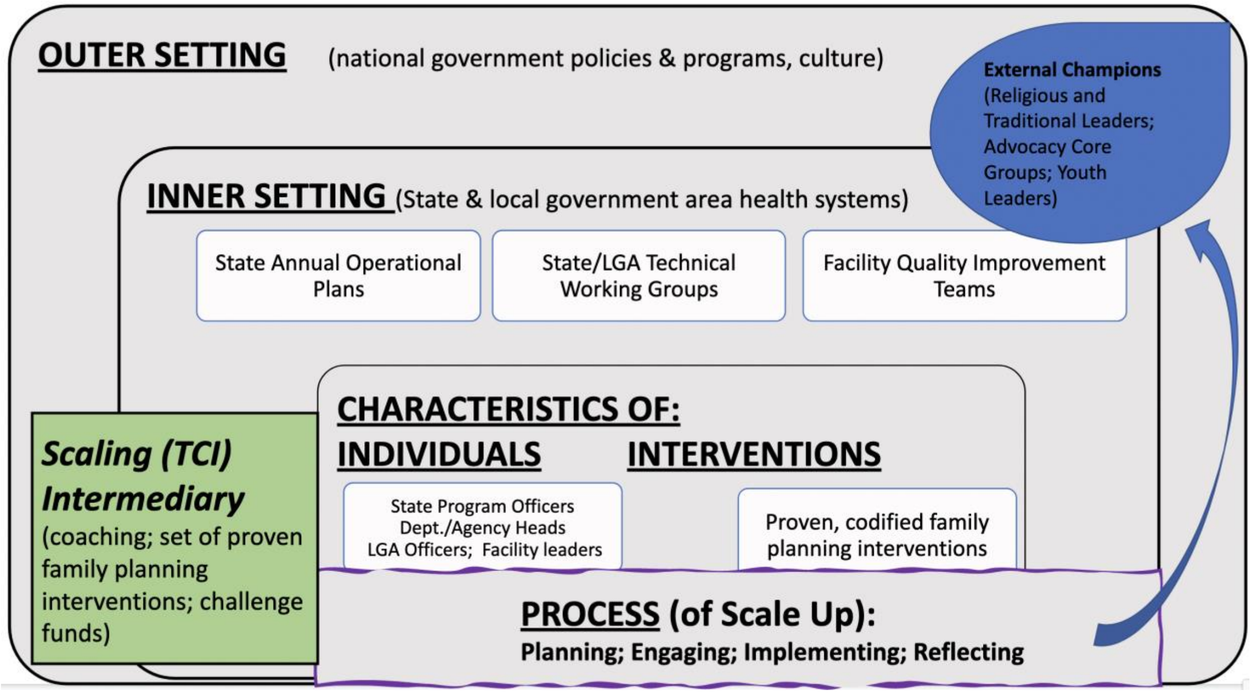

Supplement to: Ishola OD, Holcombe SJ, Ferrand A, Ajijola L, Anieto NN, Igharo V. What underlies state government performance in scaling family planning programming? A study of The Challenge Initiative state partnerships in Nigeria. *Glob Health Sci Pract*. 2023;11(Suppl 1):e2200228. https://doi.org/10.9745/GHSP-D-22-00228

Supplement 4: TCI Nigeria core high-impact interventions in the 3 states (included in a state's Annual Operational Plan)

			**State A**	**State B**	**State C**
**High-Impact Interventions**		**Brief description**	***All LGAs***	***All LGAs***	***All LGAs***
**SERVICE DELIVERY**	**Facility-Based In-Reaches** (including AY-focused inreaches)	Provision of FP services to clients mobilized from the community to a particular health facility on designated days of the week.	X	X	X
	72-hour Clinic Makeover	Swift improvement of the physical environment of clinics providing family planning services.	X	X	
	Whole Site Orientation (including an AY focus)	Facility-based orientation to improve the knowledge and attitudes of clinical and non-clinical health facility staff.	X	X	
**DEMAND GENERATION**	**Social Mobilization** *(State SBCC Committees, etc.)* **for AY and FP**	Community based sensitization activities to generate demand for FP and AYSRH services.	X	X	X
	Entertainment-Education (*radio & TV*)	Provision of FP messages and education using entertainment approaches on TV and radio.			
	Community Theater	Use of local drama troupe to dispel misconception and stimulate demand for FP demand and use.	X		
**ADVOCACY**	**Family Planning Champions** (*religious and traditional leaders, govt*)	Identification and decoration of religious and traditional leaders as champions of FP so that they may speak in favor of FP to their respective congregations.	X	X	
	Interfaith Forums	Working with religious leaders to address religious barriers to FP and sensitize their congregations on FP services.			
	Media Advocacy	Engaging media outlets to promote FP/AYSRH programs on their platforms.	X		

Supplement to: Ishola OD, Holcombe SJ, Ferrand A, Ajijola L, Anieto NN, Igharo V. What underlies state government performance in scaling family planning programming? A study of The Challenge Initiative state partnerships in Nigeria. *Glob Health Sci Pract*. 2023;11(Suppl 1):e2200228. https://doi.org/10.9745/GHSP-D-22-00228

	Advocacy at the sub-National Level (*state-level Advocacy Core Groups*)	Working with Advocacy Core Groups to foster an enabling environment, mobilize resources for the implementation of proven FP approaches and amplify voices in support of FP.			
**MANAGEMENT, GOVERNANCE and MONITORING & EVALUATION**	Strengthening Coordination Platforms	Supporting government to have functional teams that oversee program implementation, including data management.	X	X	X
	Data Use for Decision-Making	Generating real time data that can be used for program course correction.			
	Data Quality Assessment	Periodic measures used to assess data quality levels across health facilities.	X	X	X
	AYSRH data visibility and data use	Ensuring that AY specific data is visible and used to improve AYSRH program.		X	
**OTHER STAND-ALONE AYSRH HIIs**	Intergenerational dialogue	A dialogue between AYs and the older age groups within the communities to clarify values and address emerging concerns to ensure a supportive environment for FP/AYSRH programs.			
	Youth participation and engagement	Ensuring that A & Y are part of relevant leadership, management and coordination.			
